# A new proof of evidence of cysteamine quantification for therapeutic drug monitoring in patients with cystinosis

**DOI:** 10.1186/s13023-022-02540-1

**Published:** 2022-11-03

**Authors:** Martina Franzin, Silvia Rossetto, Rachele Ruoso, Rossella Del Savio, Gabriele Stocco, Giuliana Decorti, Riccardo Addobbati

**Affiliations:** 1 Institute for Maternal and Child Health-IRCCS Burlo Garofolo, 34137 Trieste, Italy; 2 Department of Medicine, Surgery and Health Sciences, University of Trieste, 34127 Trieste, Italy

**Keywords:** Cysteamine, Cystine, Cystinosis, Quantification, Therapeutic drug monitoring, LC-MS/MS

## Abstract

**Background:**

To date, measurement of intracellular cystine is used for the therapeutic monitoring of patients affected by cystinosis in treatment with cysteamine. Since this method is time and sample consuming, development of a faster method to quantify cysteamine would be extremely useful in order to help clinicians to adjust dosages of cysteamine and to define better the pharmacokinetic profile of this drug. The aim of the study was to develop a liquid chromatography tandem mass spectrometry method for the quantification of cysteamine in plasma samples and to test its applicability on plasma samples derived from patients with nephropathic infantile cystinosis in treatment with cysteamine.

**Results:**

The percentage of accuracy of the developed method varied between 97.80 and 106.00% and CV% between 0.90 and 6.93%. There was no carry over. The calibration curves were built from 2.5 to 50 µM. The limit of detection and the lower limit of quantification occurred at 0.25 and 1.25 µM respectively. Cysteamine was stable up to 2 months at -20 °C. Concentrations of cysteamine and intracellular cystine of 4 patients were in line with data previously reported.

**Conclusion:**

The proposed method showed an appropriate selectivity, specificity, linearity, sensibility, accuracy, precision and good applicability to samples.

## Background

One case per 100,000/200,000 live births suffers from cystinosis, a rare lysosomal storage disease caused by a defective membrane transport [1]. In detail, cystinosis is an autosomal recessive disease caused by several mutations in the *CTNS* gene, which encodes the carrier protein cystinosin [2]. Cystinosin is responsible for carrying cystine out of the lysosomes and mutations affecting the *CTNS* gene lead to accumulation of the amino acid inside patients’ cells resulting in the formation of crystals [3].

Although cystinosis is a monogenic disorder, three different forms can be defined: the nephropathic infantile, the nephropathic juvenile and the non-nephropathic ocular form (2). The infantile nephropathic form is the most common and severe manifestation of the disorder and is characterized by renal symptoms, such as Fanconi syndrome, and extra-renal symptoms (ocular, neurological, endocrinological, musculoskeletal and gastrointestinal symptoms) [3].

Even if cystinosis is the primary cause of inherited Fanconi syndrome in children, a differential diagnosis must be made to exclude other diseases [4]. Therefore, measurements of intracellular cystine, sequencing of *CTNS* gene and detection of the presence of cystine crystals in the cornea by microscopy are needed to diagnose the disease [4].

Among the tests available for diagnosis, measurement of intracellular cystine is actually performed in leukocytes by liquid chromatography tandem mass spectrometry (LC-MS/MS). This method has supplanted previous biochemical methods on other matrices since it is less expensive and does not require the use of radioactive materials [5, 6].

Although measurement of intracellular cystine is the most commonly used method for diagnosis and therapeutic monitoring, it requires a large amount of blood sample, expensive techniques, rapid transport of the sample and long analysis time [7].

To date, cysteamine is the only drug approved for the treatment of cystinosis. This drug is able to enter the lysosome and reacts with cystine leading to the conversion in cysteine and in a disulfide of cysteamine and cysteine; the products of the reaction can be transported out of the lysosome reducing the concentration of intracellular cystine [8, 9]. Treatment with oral cysteamine succeeds in reducing intracellular cystine content by 90% and the prognosis is much improved [2, 3]. The dosage of cysteamine based on patients’ body surface ranges from 1.30 g/m^2^ to 1.95 g/m^2^ per day divided into 4 or 2 doses depending on whether the immediate-release (Cystagon®) or the delayed-release form (Procysbi®) is administered [7, 10]. Both Cystagon® and Procysbi® are effective in lowering the concentration of intracellular cystine below the recommended value of 0.5 nmol cystine/mg of protein (expressed as hemicystine 1 nmol/mg of protein) [10, 11].

Unfortunately, patients under treatment report adverse effects, mainly gastrointestinal ones, including nausea, vomiting and abdominal pain [10]. Furthermore, several factors can interfere with cysteamine bioavailability such as intake of fatty and protein foods, causing a lower absorption, interaction with other drugs administered to manage the symptoms of the disease and, most importantly, a poor adherence to therapy [3, 7, 11, 12]. As a consequence, concentrations of intracellular cystine are not within the therapeutic range in most cases [11]. Plasma levels of cysteamine also resulted to be variable between patients and not completely defined and correlated to intracellular cystine concentrations [9, 10].

Interestingly, there is no fully standardized diagnostic method that allows the detection of cysteamine. Although there is a high interindividual variability among patients, the manuscript by Bouazza et colleagues showed a correlation between the levels of intracellular cystine and plasmatic cysteamine in patients affected by cystinosis with a pharmacokinetic model, but no development and validation of the method of detection of cysteamine was done [9]. Therefore, the scientific literature lacks new evidence to confirm the correlation already described in order to demonstrate that a fast and low-cost analytical method for the quantification of cysteamine could replace the one for the measurement of intracellular cystine in therapeutic drug monitoring (TDM). Indeed, evaluating drug concentration at regular intervals could allow to optimize therapy, increasing or reducing the standard doses to avoid inefficacy or adverse effects.

In the present study, we developed a LC-MS/MS method for the quantification of cysteamine in plasma samples and tested its applicability on 4 patients with nephropathic infantile cystinosis in treatment with this drug. This analytical method could be useful in the future to define a specific interval of plasma concentrations of cysteamine related to levels of intracellular cystine in the therapeutic range and could be implemented easily into the laboratory routine allowing a more efficient and faster TDM.

## Results

### Method development

#### LC conditions

Poroshell Agilent 120 EC-C8 150 mm×2.1 mm, 4 μm (Agilent Technologies, Santa Clara, CA, USA) was chosen thanks to its capability to obtain higher, narrower and symmetrical peaks compared to the ones obtained with the other columns tested. In order to perform the analytes’ separation, the stationary phase was eluted with mobile phase A (water with 0.15% formic acid and 5 mM ammonium formate) and mobile phase B (acetonitrile:water 95:5 with 0.15% formic acid and 5 mM ammonium formate). The mobile phases were delivered in gradient mode as described in Table 1 in a total run of 14 min at a flow rate of 0.4 mL/min.

**Table 1:** Optimized gradient for LC-MS/MS analysis for the quantification of cysteamine in plasma.

Time	% Eluent A	% Eluent B	Flow
0.0	99	1	0.4 mL/min
6.0	80	20	0.4 mL/min
6.5	0	100	0.4 mL/min
8.5	0	100	0.4 mL/min
8.6	99	1	0.4 mL/min
14	99	1	0.4 mL/min

**Fig. 1:**
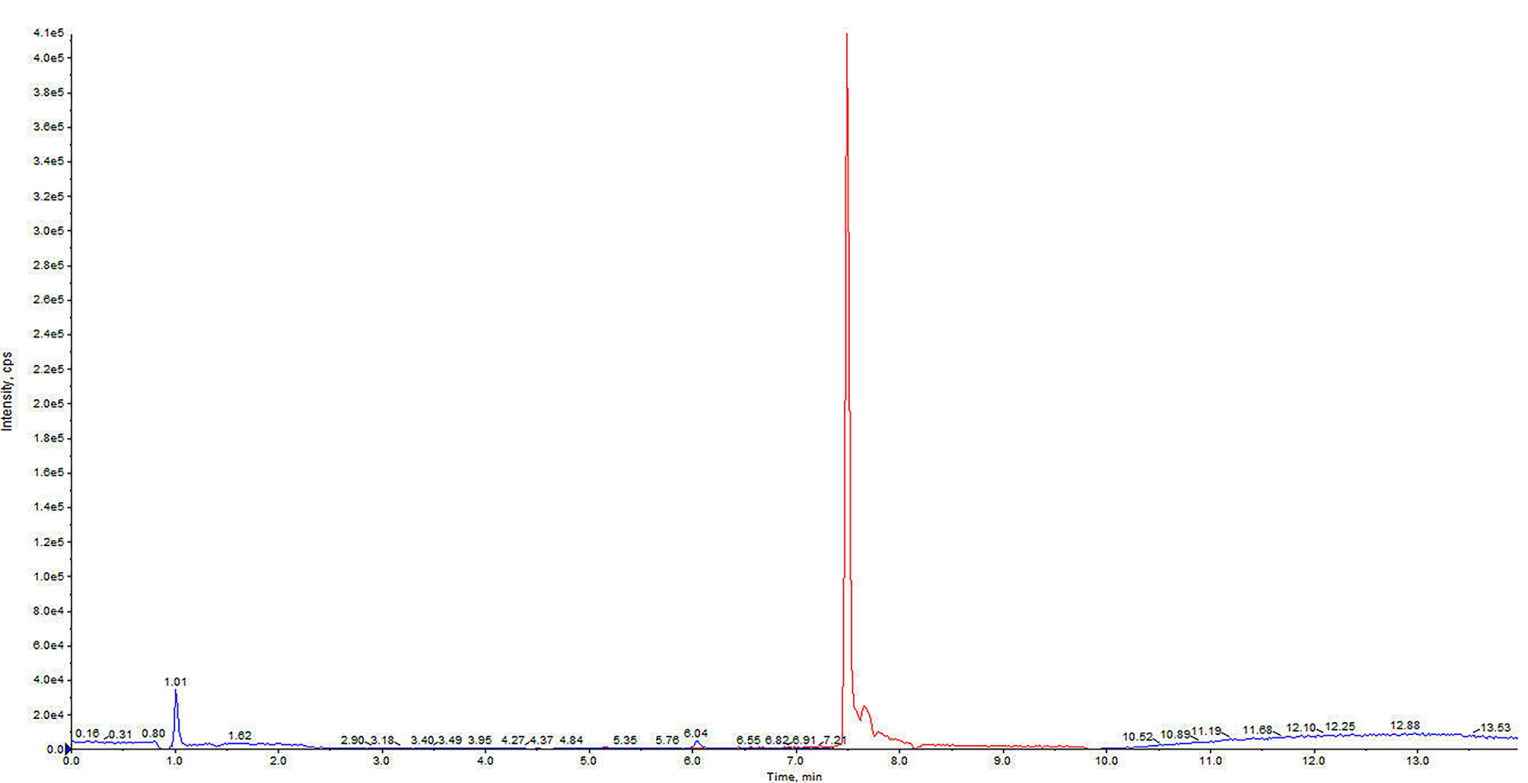
Chromatogram showing the retention times of the analyte cysteamine (retention time = 1.01; blue line) and the IS D6-cystine (retention time = 7.49; red line).

During the analysis, the samples were kept at 4 °C in the autosampler and the column oven was set at 30 °C. The injection volume was 3 µL. As shown in Fig. 1, the retention time of the analyte cysteamine and of the IS D6-cystine was 1.01 and 7.49 min respectively.

#### MS conditions

The *m/z* ratios of the precursor ions of cysteamine and of the IS used, D6-cystine, were already known from scientific literature; instead, the *m/z* ratios of product ions of each compound were set up through the product ion scan acquisition mode. Quantitative analysis was achieved with multiple reaction monitoring (MRM) scan mode in positive ionization. Among the three transitions selected for cysteamine, *m/z* = 61.000 was used to quantify and the others to confirm the analyte. Among the two transitions selected for D6-cystine, *m/z* = 131.300 was used to quantify and the other to qualify the IS. The compound dependent MS parameters were also optimized and were specified in Table 2 together with the *m/z* ratios

**Table 2:** Optimized MS parameters of the LC-MS/MS method related to the analyte or to the IS. The first transition of the product ion was chosen for the quantification and the other for the confirmation of the compound. DP declustering potential; EP entrance potential; CE collision energy; CXP collision cell exit potential.

Compound	Precursor ion (*m/z*)	Product ion (*m/z*)	Dwell time (ms)	DP (V)	EP (V)	CE (V)	CXP (V)
Cysteamine	77.900	61.000	200	20.000	7.900	16.000	10.000
	77.900	35.000	200	20.000	7.900	31.000	16.000
	77.900	27.000	200	20.000	7.900	36.000	12.000
D6-cystine	359.300	131.300	100	25.000	8.000	20.000	9.000
	359.300	211.300	100	20.000	8.000	20.000	9.000

The source dependent MS parameters were fixed as recommended by the manufacturer: curtain gas (CUR) 30 psi, collision gas (CAD) medium, ion spray voltage (ISV) 5200 V, source temperature (TEM) 450 °C, ion source gas1 (GS1) 50 psi, ion source gas2 (GS2) 55 psi and interface heater on

#### Selectivity and specificity

Selectivity of the analytical method was assessed analyzing plasma samples from different healthy volunteers. No other compound, except for the analyte and IS, was detected in the run; therefore, the method proved to have specificity towards cysteamine and the IS used.

#### Linearity

Calibration standards (2.5, 5, 10, 25, 50 µM) were analyzed in triplicate in three different days. The calibration curve was constructed plotting the areas of each concentration level corrected with the IS area versus the nominal values. QCs were also analyzed in each analytical run and the concentration were calculated comparing the ratio between the analyte peak area and the IS peak area with the calibration curve relation. Table 3 showed three calibration curves and the calculated concentration of the QCs analyzed with the same analytical runs.

**Table 3:** Peak areas and peak heights of analyte and IS referred to the calibrators and to the QCs with the corresponding calculated concentration. CAL calibrators. NA not available. Cps counts per second calculated concentration and accuracy

Standard	Analyte peak area (counts)	Analyte peak height (cps)	IS peak area (counts)	IS peak height (cps)	Nominal concentration (µM)	Calculated concentration (µM)
*1°CALIBRATION CURVE R*^2^ *= 0.9998 y = 0.00532x-0.00858*						
CAL 0	0.0	0.0	1.77*10^6^	6.44*10^5^	0.0	0.0
CAL1	2.45*10^4^	7.89*10^3^	4.91*10^6^	1.91*10^6^	2.5	2.67
CAL2	4.43*10^4^	1.86*10^4^	2.43*10^6^	9.07*10^5^	5	5.15
CAL3	1.55*10^5^	5.87*10^4^	3.45*10^6^	1.39*10^6^	10	9.94
CAL4	2.80*10^5^	1.05*10^5^	2.30*10^6^	8.70*10^5^	25	24.5
CAL5	6.17*10^5^	2.33*10^5^	2.38*10^6^	8.96*10^5^	50	50.2
QC I	7.52*10^4^	2.75*10^4^	6.04*10^6^	2.33*10^6^	4	4.06
QC II	1.96*10^5^	6.86*10^4^	9.81*10^5^	3.98*10^5^	40	39.1
*2° CALIBRATION CURVE R*^2^ *= 0.9995 y = 0.00475x + 0.00569*						
CAL 0	0.0	0.0	7.97*10^5^	2.51*10^5^	0.0	0.0
CAL1	4.92*10^4^	1.58*10^4^	2.73*10^6^	9.18*10^5^	2.5	2.60
CAL2	3.03*10^4^	1.20*10^4^	9.47*10^5^	3.14*10^5^	5	5.52
CAL3	1.16*10^5^	4.72*10^4^	2.08*10^6^	7.23*10^5^	10	10.6
CAL4	1.93*10^5^	6.62*10^4^	1.53*10^6^	5.94*10^5^	25	25.2
CAL5	4.40*10^5^	1.46*10^5^	1.81*10^6^	6.40*10^5^	50	49.7
QC I	9.41*10^4^	3.48*10^4^	3.78*10^6^	1.52*10^6^	4	4.05
QC II	1.96*10^5^	6.86*10^4^	1.01*10^6^	4.05*10^5^	40	39.5
*3° CALIBRATION CURVE R*^2^ *= 0.9993 y = 0.00535x-0.000279*						
CAL 0	0.0	0.0	6.89*10^5^	2.41*10^5^	0.0	0.0
CAL1	2.69*10^4^	7.73*10^3^	1.94*10^6^	8.48*10^5^	2.5	2.65
CAL2	4.53*10^4^	1.49*10^4^	1.64*10^6^	5.64*10^5^	5	5.23
CAL3	1.09*10^5^	3.78*10^4^	2.00*10^6^	7.39*10^5^	10	10.3
CAL4	1.75*10^5^	5.69*10^4^	1.39*10^6^	5.06*10^5^	25	23.6
CAL5	3.68*10^5^	1.36*10^5^	1.43*10^6^	5.27*10^5^	50	50.6
QC I	5.61*10^4^	1.89*10^4^	2.79*10^6^	1.04*10^5^	4	3.82
QC II	2.76*10^5^	9.92*10^4^	1.17*10^6^	4.06*10^5^	40	44.2

#### Sensibility

**Fig. 2:**
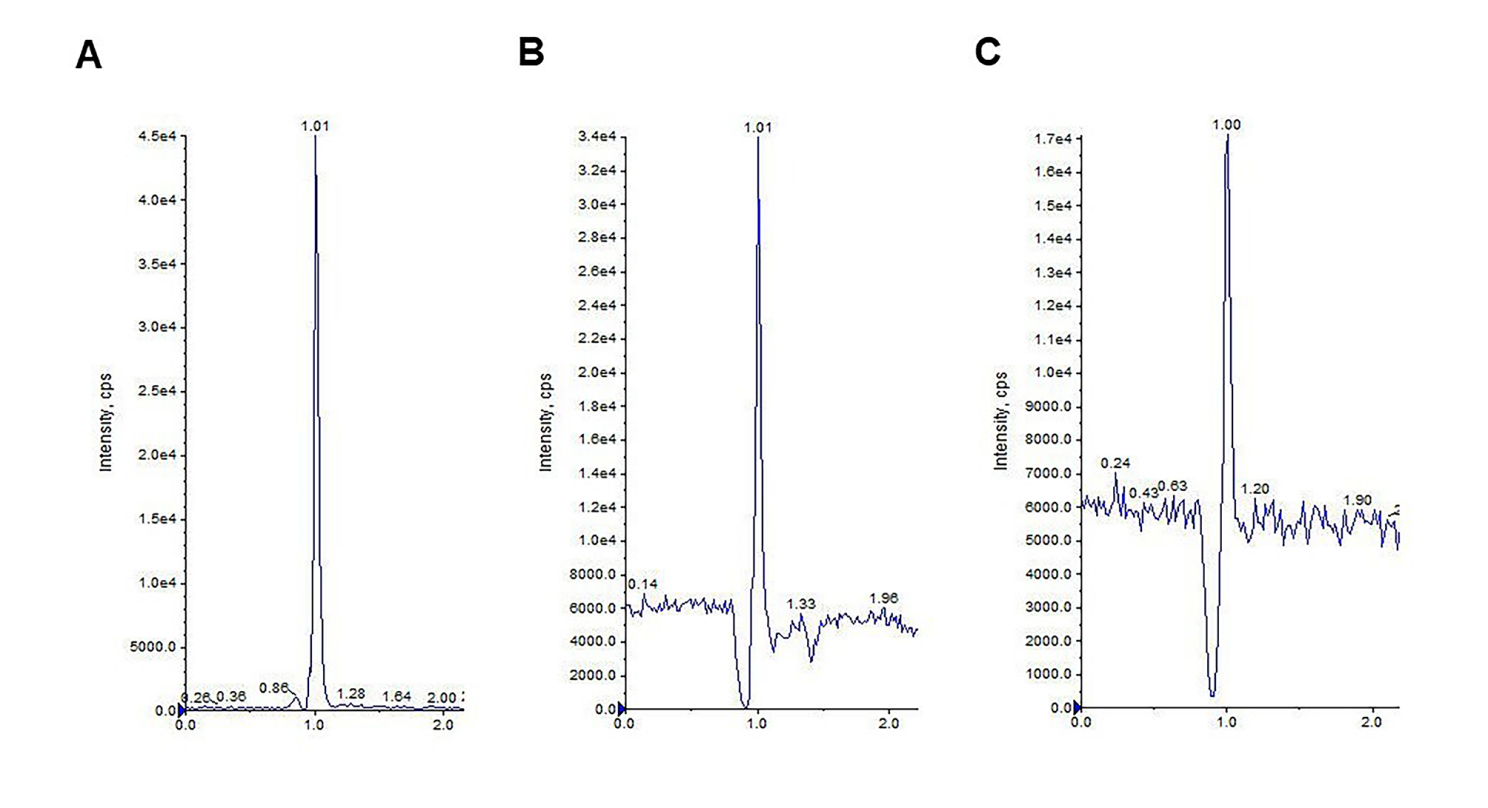
Chromatograms obtained after injecting calibrator 1 (A), dilution 1:2 of calibrator 1 corresponding to LOQ (B) and dilution 1:10 of calibrator 1 corresponding to LOD (C)

Dilutions of the calibrator with the lowest concentration (2.5 µM) were carried out in order to identify the lowest concentration of analyte that the method is able to detect, corresponding to limit of detection (LOD), and able to quantify, corresponding to LLOQ. As reported in Table 4, testing the dilution 1:2 (1.25 µM), the concentration of cysteamine resulted to be detectable and quantifiable with an accuracy of 105%; this concentration can be considered the LLOQ. Instead, testing the dilution 1:10 (0.25 µM), the signal was detectable but was not quantified with a sufficient accuracy and precision; therefore, it was defined as the LOD. Chromatograms related to calibrator 1, LOQ and LOD respectively are shown in Fig. 2.

**Table 4:** Peak areas and peak heights of analyte and IS referred to the calibrator CAL1 (2.5 µM) and to its dilutions 1:2 (1.25 µM) and 1:10 (0.25 µM) with the corresponding calculated concentration and accuracy. ND not detectable. Cps counts per second.

Sample	Analyte peak area (counts)	Analyte peak height (cps)	IS peak area (counts)	IS peak height (cps)	Nominal concentration (µM)	Calculated concentration (µM)	Accuracy (%)
CAL1	1.71*10^5^	7.94*10^4^	5.10*10^5^	1.89*10^5^	2.60	2.50	104
CAL 1 (1:2)	4.70* 10^4^	1.91*10^5^	3.11*10^5^	1.12*10^5^	1.31	1.25	105
CAL 1 (1:10)	0.0	0.0	3.87*10^5^	1.35*10^5^	ND	0.25	ND

#### Accuracy and precision

Accuracy and precision were evaluated both for calibrators and QCs; in particular QCs’ concentrations were calculated on the basis of calibration curve. Table 5 shows results of inter-day accuracy, and precision, represented by the coefficient of variation (CV%). The percentage of accuracy varied between 97.80 and 106.00% and CV% between 0.90 and 6.93%.

**Table 5:** Inter-day accuracy and precision of calibrators and QCs.

Standard	Nominal concentration (µM)	Accuracy (%)	CV (%)
CAL 1	2.5	105.66	1.37
CAL 2	5	106.00	3.67
CAL 3	10	102.80	3.21
CAL 4	25	97.80	3.28
CAL 5	50	100.13	0.90
QC I	4	99.46	3.41
QC II	40	102.53	6.93

#### Carry over

**Fig. 3:**
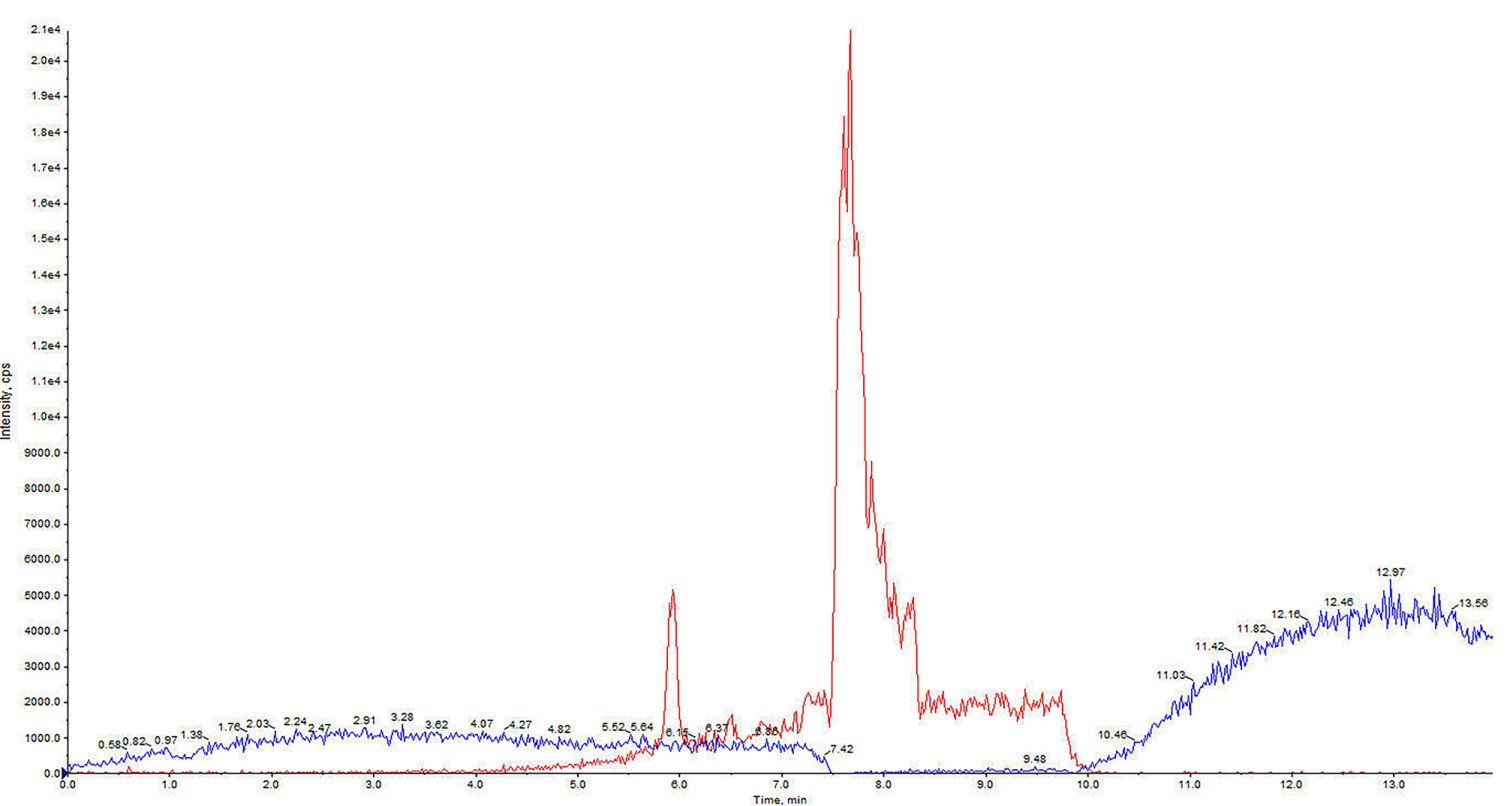
Chromatogram obtained after injecting a blank sample after the calibrator with the highest analyte concentration. The blue and red lines consist in respectively the signal along the chromatogram derived from the transition of cysteamine and of IS used to quantify

Carry over was assessed by injecting blank samples after the calibrator with the highest analyte concentration and, as shown in Fig. 3, the signal derived was not greater than 20% and 5% of that of the analyte and of IS respectively.

#### Stability of cysteamine

The stability of the 100 µM cysteamine in plasma was evaluated using the working solutions for the preparation of calibrators and QCs after 1, 2 and 3 months from its preparation. The solution was stored at -20 °C and was found to be stable up to 2 months at these conditions.

### Application of the method to samples

Cysteamine and intracellular cystine were measured in 4 patients suffering from nephropathic infantile cystinosis in order to test the applicability of the analytical method. As shown in Tables 6 and 7, the concentrations of plasmatic cysteamine and of intracellular cystine were in line with those expected for patients after 6 h since the oral administration of cysteamine bitartrate.

Our results confirmed previously reported data: cysteamine succeeds in lowering the concentrations of intracellular cystine below the recommended value of 1 nmol of hemicystine/mg of protein [9–11].

**Table 6:** Measurements of plasmatic concentrations of cysteamine in 4 patients suffering from nephropathic infantile cystinosis after 6 h since the administration of cysteamine bitartrate

Patient	Cysteamine concentration (µM)
A	2.30
B	1.77
C	3.92
D	1.70

**Table 7:** Measurements of intracellular concentrations of cystine in 4 patients suffering from nephropathic infantile cystinosis after 6 h since the administration of cysteamine bitartrate

Patient	Cystine concentration (nmol hemicystine/mg of protein)
A	0.47
B	0.50
C	0.67
D	0.50

Pearson correlation test did not evidence a linear relationship between the cystine and cysteamine concentrations (*p* = 0.142).

**Fig. 4:**
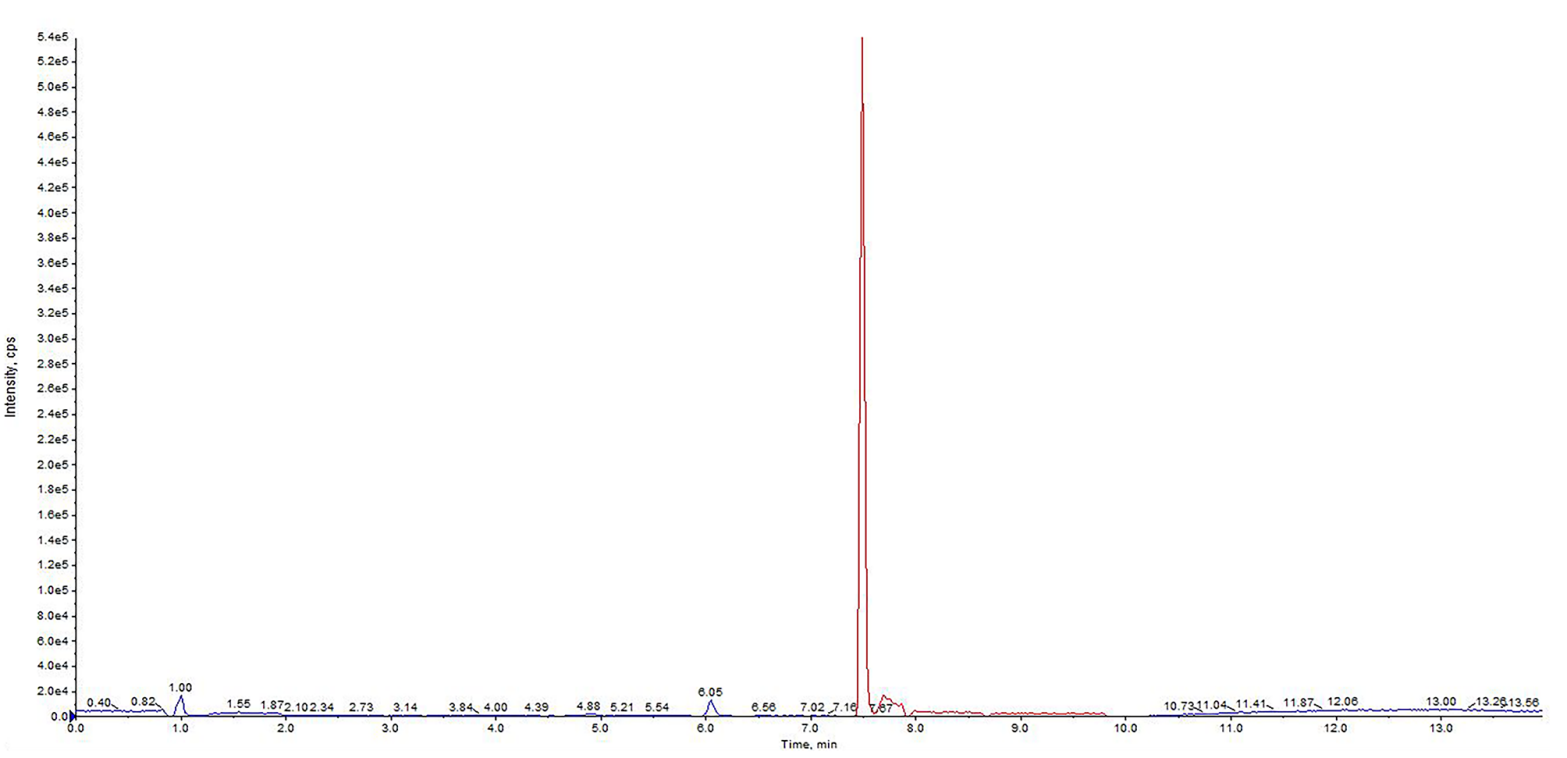
Chromatogram obtained after injecting a sample derived from a healthy individual not affected by cystinosis and not undergoing cysteamine treatment. The blue and red lines consist respectively in the signal derived from the transition of cysteamine and of IS.

Furthermore, we tested also a healthy individual not affected by cystinosis and not undergoing treatment with cysteamine, as negative control. As shown in Fig. 4, no peak of the analyte cysteamine distinguishable from background noise was evidenced. The only peak present was that of the IS.

## Discussion

Measurement of intracellular cystine is actually used both as diagnostic tool and as therapeutic monitoring in patients with cystinosis. Despite its proven advantages, it requires a large amount of blood sample, expensive techniques, rapid transport of the sample and especially long analysis time (7). Therefore, it would be necessary to have a faster method to allow therapeutic monitoring, allowing clinicians to adjust dosages of cysteamine, the drug used in this disease.

To date, there is no fully standardized diagnostic LC-MS/MS method for the detection of cysteamine. Several methods have been described in the scientific literature to quantify cysteamine in biological samples such as enzymatic tests, ion exchange column chromatography, high-performance liquid chromatography with fluorescence or UV detection, gas chromatography with flame ionization and photometric detection [12]. Bouazza et colleagues proposed also a LC-MS/MS method for cysteamine detection but the method was not fully developed and validated [9].

In the present study we developed a quick, low-cost and low sample volume LC-MS/MS method for the quantification of cysteamine in plasma of patients suffering from cystinosis. The method showed an appropriate selectivity, specificity, linearity, sensibility, accuracy and precision for the quantification of cysteamine. In detail, the proposed LC-MS/MS method requests only 3 µL of the analyte suspension and the total run lasts 14 min. It shows a percentage of accuracy between 97.80 and 106.00% and CV% between 0.90 and 6.93% and presents no carry over. The calibration curves were built from 2.5 to 50 µM and covers the range of concentration noticed previously in patients suffering from cystinosis in treatment with cysteamine [9]. The LOD occurs at 0.25 µM and the LLOQ at 1.25 µM.

Contrary to previous studies, we tested also the stability of cysteamine in the matrix and found out that it is stable in plasma at -20 °C up to 2 months [9, 10]. Moreover, D6-cystine as IS was used for the first time in cysteamine quantification: this choice is really convenient since it is the same IS for the LC-MS/MS method for the quantification of intracellular cystine.

In order to test the applicability of the method for the quantification of cysteamine to samples, we analyzed both intracellular content of cystine and plasma concentrations of cysteamine in 4 patients with nephropathic infantile cystinosis after 6 h since the administration of the drug. As previously reported [9–11], cysteamine succeeded in lowering the concentrations of intracellular cystine below the recommended value of 1 nmol of hemicystine/mg of protein. Moreover, the measurements of the cysteamine and of intracellular cystine were in line with the scientific literature suggesting that the proposed LC-MS/MS method could be applied to other studies in this field and validated [9, 10].

According to the studies of Bouazza et al. (9) and van Stein et al. [10], plasma concentrations of cysteamine seem to be variable between patients; this could be since the cohorts taken in consideration differ greatly by age (0.4–36 years and 12.3–33.3 years respectively) and it is well known that pharmacokinetic parameters change depending on the﻿ age [13]. Therefore, to date, there is no reference value for plasma levels of cysteamine associated to inefficacy of therapy or to the presence of adverse effects although the frequency of these is high [10, 11].

Unfortunately, no correlation was found between the measurements of intracellular cystine and cysteamine concentrations maybe because of the small number of the patients tested. It would be necessary to analyse a cohort with a larger number of patients in order to assess the real relationship between the concentrations of intracellular cystine and cysteamine.

## Conclusion

In the present study we developed a LC-MS/MS method for the quantification of cysteamine that showed an appropriate selectivity, specificity, linearity, sensibility, accuracy and precision and we applied the method to 4 patients suffering from nephropathic infantile cystinosis after 6 h from the administration of cysteamine. The encouraging results and its good applicability could lead to the use of this method to new research in this field in order to acquire new evidence regarding a reference value for plasma concentrations of cysteamine associated with inefficacy of therapy or the presence of adverse effects in pediatric patients with nephropathic infantile cystinosis. Other future perspectives, although ambitious, aim at replacing the non-convenient direct measurement of intracellular cystine in the laboratory diagnostic routine.

The limits of the study concern the small number of the patients tested for the applicability, due to the fact that the disease is rare, and the lack of clinical information about the patients tested. As already mentioned, future perspectives would be to increase the number of cystinosis patients under treatment in order to assess the relationship between the concentrations of intracellular cystine and cysteamine.

As a whole, this study would be a new proof of evidence of cysteamine quantification for TDM in patients with cystinosis.

## Methods

### Quantification of cysteamine

#### Chemicals and materials

3 N HCl-butanol, acetonitrile, ammonium formate, cysteamine, the internal standard (IS) D6-cystine, dithiothreitol (DTT), ethylenediamine tetraacetic acid (EDTA), formic acid, LC-MS grade water, NaOH, N-ethylmaleimide (NEM), methanol and sulfosalycilic acid (SSA) were purchased from Sigma-Aldrich (St. Louis MO, USA).

#### Stock and working solutions

Stock solutions of 1 mM cysteamine were prepared by dissolving 11.3 mg of analyte in 100 mL of water. The working solution of 100 µM cysteamine was prepared from the stock solution diluting 1:10 in plasma. Stock solutions of 1 mM D6-cystine were prepared by dissolving 6 mg of internal standard in a solution of 0.4 mg/mL BSA and adding 60 µL of HCl 37%. The working solution of 10 µM D6-cystine was prepared from the stock solution diluting 1:100 in plasma. DTT solution 0.1 M, used as reducing agent, was prepared dissolving 15.4 mg of reagent in 1 mL of 5 mM NaOH. EDTA 0.2 M was prepared dissolving 3.72 g in 50 mL of water. SSA 12% was prepared dissolving 12 g in 100 mL of water. NEM solution 0.65 mg/mL, used as alkylating agent, was prepared dissolving 2.6 mg in 4 mL 0.9% NaCl. After being prepared, stock and working solutions were spiked and stored at -20 °C. Plasma used to dilute the solutions was obtained from a plasma pool prepared mixing samples from different healthy individuals with the same age of the population of reference allowing to have a representative matrix. The plasma pool was stored also at -20 °C.

#### Calibration and quality control samples

Calibration standards and quality controls (QCs) samples were prepared spiking the plasma pool with different amount of the 100 µM cysteamine working solution.

Calibration curve was built from 2.5 to 50 µM on the basis of the therapeutic range expected [9]. Taking into account the working range, the chosen QCs were 4 and 40 µM and were prepared from a stock solution different from the one used for the calibrators to avoid biased estimations. In all the calibrators and the QC samples, the IS D6-cystine was added at the beginning of the extraction procedure. The preparation of calibrators and QCs was the same reported for sample preparation.

#### Plasma sample collection

Venous blood samples (4 mL) were collected into sodium heparin tubes from 4 patients suffering from nephropathic infantile cystinosis after 6 h from the administration of cysteamine bitartrate and just before the following administration. Patients received the drug as capsule every 6 h. Blood sample was transferred to a 15 mL centrifuge tube. EDTA 0.2 M was added and the sample was gently mixed. Plasma was isolated centrifuging for 15 min at 1200 xg and stored at -20 °C until the LC-MS/MS analysis.

#### Sample preparation

On the day of the analysis, 100 µL of sample were added to a mixture of 20 µL 10 µM D6-cystine, 10 µL of DTT solution, 10 µL of NEM solution and 10 µL of 12% SSA. After vortexing and centrifuging for 1 min at 2500 xg, 150 µL of methanol were added. After vortexing and centrifuging for 5 min at 15,000 xg, the supernatant was transferred in a new tube and evaporated under nitrogen stream. One hundred µL of 3 N HCl-butanol were added to the pellet to allow derivatization of the IS and then the sample was incubated for 30 min at 65 °C. After vortexing and centrifuging for 1 min at 2500 xg, the supernatant was again evaporated under nitrogen stream and the analyte was resuspended in 150 µL of water.

### Quantification of intracellular cystine

Intracellular cystine was also measured in leukocytes isolated from the same venous blood samples used for cysteamine quantification with a LC-MS/MS method previously reported in scientific literature, adapted and validated at IRCCS Burlo Garofolo Hospital and used in diagnostics [5]. Protein quantification was also performed and the results were expressed in nmol of hemicystine/mg of protein.

### Instrumentation

The chromatographic system consisted of a SCIEX ExionLCTM AD (AB Sciex, Foster city, CA, USA) and was coupled with the mass spectrometer SCIEX Triple QuadTM 6500 + LC-MS/MS System (AB Sciex, Foster city, CA, USA) operated in electrospray ionization mode. Chromatograms were recorded and mass spectrometer parameters were optimized with the software Analyst 1.7 (AB Sciex, Foster city, CA, USA).

### Data analysis

Regarding the LC-MS/MS analysis, data analysis was performed with the software Analyst 1.7. 1/χ2 statistical weight was applied to the calibration curve.

## Data Availability

The data supporting the findings of the article is available upon request to the corresponding author.

## List of abbreviations

LC-MS/MS: liquid chromatography tandem mass spectrometry.
IS: internal standard.
TDM: therapeutic drug monitoring.
DTT: dithiothreitol.
EDTA: ethylenediamine tetraacetic acid.
NEM: N-ethylmaleimide.
SSA: sulfosalycilic acid.
QCs: quality controls.
MRM: multiple reaction monitoring.
DP: declustering potential.
EP: entrance potential.
CE: collision energy.
CXP: collision cell exit potential.
CUR: curtain gas.
CAD: collision gas.
ISV: ion spray voltage.
TEM: source temperature.
GS1: ion source gas1.
GS2: ion source gas2.
CAL: calibrators.
NA: not available.
LOD: limit of detection.
LLOQ: lower limit of quantification.
CV: coefficient of variation.
